# Gamification of Cognitive Behavioral Therapy Homework: Therapist Concept Mapping Approach

**DOI:** 10.2196/50923

**Published:** 2026-04-30

**Authors:** Ho Ming Lau, Patricia van Oppen, Heleen Riper

**Affiliations:** 1 Department of Clinical Psychology, Faculty of Behaviour and Movement Science, Vrije Universiteit, Van der Boechorststraat 7, Amsterdam, 1081 BT, The Netherlands, 31 618412735; 2 Department of Psychiatry, Amsterdam Public Health Research Institute, Amsterdam UMC, Vrije Universiteit, Amsterdam, The Netherlands

**Keywords:** gamification, cognitive behavioral therapy, CBT, concept mapping, game design elements, depression, anxiety, mental health, game-based, homework, motivation, adherence, compliance

## Abstract

**Background:**

Greater homework adherence in cognitive behavioral therapy (CBT) is associated with positive treatment outcomes. However, the problems emerging from CBT homework use are common and affect adherence. In recent years, gamification has been explored to increase intervention adherence, but not yet in relation specifically to homework assignments.

**Objective:**

In this study, the aim was to gain a better understanding of obstacles to CBT homework and the use of gamification to overcome these.

**Methods:**

Concept mapping, a method to organize related information visually, was used in this study. For the 1-day face-to-face concept mapping session, 7 therapists (32 to 55 y, 6 females) participated and generated items based on 2 focal questions of interest. The generated items were grouped on perceived similarity, and each individual item was rated on (1) severity and difficulty (focal question 1) and (2) importance, acceptance by therapist, and acceptance by patient (focal question 2). The item groups on perceived similarity were inserted into computer software. Based on multidimensional scaling and hierarchical cluster analyses, item clusters were generated by the computer software and were presented to the therapists. The therapists were asked for their preference for the number of items a cluster should contain.

**Results:**

Through brainstorming, the therapists collectively generated a list of 29 possible reasons for not doing homework by patients. In the same manner, a list of 38 game design elements that could help patients make CBT homework was generated. External factors (eg, no time due to crisis situations) and lack of motivation (eg, not aspiring to a therapy goal) were perceived as the most important reasons for patients not to do homework. External and symptoms-unrelated internal factors were considered by therapists as the most difficult for patients to change for improved homework adherence. The game design elements, facilitation, and rewards were rated as most important to help patients do homework. These elements were also seen as most accepted by therapists.

**Conclusions:**

Facilitation of doing homework and rewards seem to have the potential to tackle some of the external factors and lack of motivation to make CBT homework that patients could have. Conclusions were limited by the small number of participating therapists. Future research is needed on the effects of specific game design elements, the number of these elements, their combinations, and patients’ preferences.

## Introduction

Homework in the context of cognitive behavioral therapy (CBT) for depression is assignments that clients are instructed to do between therapy sessions to help them make progress toward therapeutic goals [1]. The rationale behind the use of homework is that a therapy session represents only a small fraction of available time in a person treated for depression, and such homework assignments provide a means to enhance mastery of newly learned coping strategies, facilitate generalization of skills to novel situations, increase self-efficacy, and ultimately reduce vulnerability to relapse [2]. Homework is an important component of CBT because it can predict therapy outcome [3-5]; studies have shown that greater homework adherence is associated with positive treatment outcomes for depression [1,3,6,7].

The use of homework CBT can therefore be a great force in overall improvement, but also a bottleneck at the same time. Literature shows that problems emerging from CBT homework use are common and affect adherence to homework [8,9]. These problems can be patient, task, or therapist-related [10,11]. From the perspective of the patient, doubting their abilities to finish the task or not being motivated can be barriers [9,12]. Especially, not having motivation, one of the symptoms of depression, to do the task is strongly linked to lower homework adherence [12]. Task difficulty can be an obstacle to adherence as well. Therapists rarely modify the assignments to match with patients’ abilities, but rather explain goals and background of the tasks. According to Borgart and Kemmler [13], 60% of the behavioral therapists in their study reported that homework assignments were frequently modified by the patient. Also, therapists’ lack of giving written instructions during assignment of homework is correlated with lower adherence [9]. As can be seen, CBT homework has barriers; removing those barriers can improve therapy adherence and thus be a mediator to a more successful outcome of the treatment.

Several attempts have been made to try to increase CBT homework adherence using technology, namely computerized CBT or internet-based CBT [14,15]. Willner-Reid et al [16] used electronic devices to remind patients to complete their homework. Also, the use of digital worksheets between sessions to discover potential barriers was explored [17]. Several mobile apps have been developed to support homework completion [18-24], and an internet-based support system has also been developed [25]. In guided online therapy, the therapist or coach plays an important role by providing feedback on homework assignments [26]. Also, the role of conversational agents in supporting CBT-based treatment has been explored [27]. Some of the used technologies show positive results on usability and effectiveness [18,20], while others still need to be tested on effectiveness.

In recent years, a new facilitator that can help increase homework adherence has been introduced—gamification [28,29]. Gamification is the use of game principles in a nongame context [30]. Using gamification in CBT means adding game principles around homework assignments. For example, giving rewards for completing homework. This can be done digitally on a computerized CBT or internet-based CBT platform (eg, giving an achievement emblem after completing a homework assignment) or nondigitally (eg, giving a real flower as a reward for doing homework). For example, the CBT-based gamification app SuperBetter was shown to reduce depression symptoms [31]. In this app, goals, mood-boosting activities, and obstacles are named quests, power-ups, and battle bad guys, respectively. Users receive points and level-up through the activities. Adding CBT elements to games is also possible. For example, the playable activities in the serious game SPARX (The University of Auckland) [32,33] are based on CBT assignments, showing that SPARX appears to be a promising treatment for students with depression symptoms. Although evidence is limited, current literature shows that exploiting game characteristics to change patients’ behavior has potential [8,34].

In a systematic review, the effect of gamification features on adherence to online mental health interventions primarily based on CBT was studied [35]. The adherence was measured for the intervention in general and not homework-specific. No differences in therapy adherence were found between intervention and control groups when only 1 gamification feature was used. However, when additional features were added, a higher significant mean adherence in favor of the intervention group was found.

Some questions remain unanswered by the current literature. How could game design elements help improve CBT homework adherence? For gamification to have a full impact in interventions, such as homework assignments, it is necessary to select the key game design elements [28] and design the intervention on well-founded theories [36]. In order to get more clarity about the different components, therapists in this study were asked questions about possible obstacles to doing homework and the potential of game design elements in homework in a systematic way.

In this study, we aimed to (1) obtain a therapist-generated list of possible obstacles that patients face in making CBT homework, (2) obtain a therapist-generated list of game design elements that could help motivate patients to do homework, and (3) gain a better understanding of the potential of how gamification can be applied best in CBT homework.

## Methods

### Overview

We used the concept mapping methodology, originally introduced by Trochim [37], to identify (1) possible reasons that patients have according to therapists for not complying (fully) with the CBT homework and (2) game design elements that could help patients to comply more with CBT homework. According to Trochim and Kane [38], concept mapping is a methodology to organize information in a visual way. It combines specific analysis and data interpretation methods to produce maps so that interrelations in the information can be seen.

The concept mapping methodology according to Kane and Trochim [39] consists of six phases: (1) preparation, (2) generation, (3) structuring, (4) representation, (5) interpretation, and (6) utilization. In the (1) preparation phase, focal areas and criteria for participant selection are determined. In the (2) generation phase, selected participants brainstorm on the focal question and generate a list of items that will be used in the next phase. During the (3) structuring phase, participants individually organize the generated items in groups of perceived similarity. Thereafter, each item is rated on 2 or more domains. The representation phase (4) consists of entering the sorted and rated data into concept mapping software to make visual representations (concept maps). In the (5) interpretation phase, participants analyze the data qualitatively and discuss the results collectively. In the final phase, (6) utilization, participants discuss how the results relate to the focal question.** **

### Phase 1: Preparation

#### Overview

The researchers outlined three study goals: (1) to obtain a therapist-generated list of possible obstacles that patients face in making CBT homework, (2) to obtain a therapist-generated list of game design elements that could help motivate patients to do homework, and (3) to gain a better understanding of the potential to use gamification in CBT.

#### Focal Questions

The researchers prepared two focal questions:

1. What are the possible reasons for patients’ failure to complete their homework?
2. What game design elements could be important to help clients do their homework better or more frequently?

#### Participants

During the preparation step, the researchers determined the participants’ inclusion criteria and the recruitment process of the participants. A group of seven CBT therapists (32 to 55 y, 6 females) working with patients with depressive and anxiety disorders was identified and invited to participate in the concept mapping meeting. They were employed by GGZ inGeest, a large mental health organization providing specialized mental health care in Amsterdam, the Netherlands. One week before the concept mapping meeting, participants received a book on gamification [40] to give them a context for the meeting. The participants were encouraged to read the first chapter of the book, which gave a general introduction to gamification.

#### Facilitator

Phases 2 (*generation*) and 3 (*structuring*) were led by an external facilitator to ensure objectivity. It was important not to steer the participants in a certain direction because of personal experience or perspective. The facilitator did not have previous experience with gamification or CBT. The facilitator was given a general introduction to gamification by the researcher (HML) before the start of phase 2. The researcher also instructed the facilitator to help the participants stay in the context but not to steer them.

### Phase 2: Generation

The concept mapping meeting was held on March 31, 2017. Before the generation phase started, the facilitator explained the purpose and guidelines of the brainstorm session. Next, the researcher (HML) gave a general introduction to gamification, after which the generation process began.

First, the participants were asked to individually generate items based on focal question 1. The items were written down by the participants on their own sheet of paper. After this, the participants brainstormed on the same matter as a group. To maximize participation, therapists named one individually generated item, taking turns. This continued till all the items on the sheets of the therapists’ papers were named. When triggered by others’ items, all participants were allowed to name new items. When no new items were generated, the group brainstorming ended. All items were entered into the computer immediately and projected on a large screen so that every participant could see all the generated items in order to prevent duplicate items. The same procedure was followed for focal question 2: What game design elements could be important to help clients do their homework better or more frequently?

### Phase 3: Structuring

#### Sorting

During the same concept mapping meeting, each participant also received a set of cards with all generated items by the total group from the previous phase. On each card, one item was printed. Participants were asked to sort the generated items by grouping them into piles that made sense to them [41,42]. The facilitator instructed the participants that each item could be placed in 1 pile only. Also, each pile had to contain at least 2 items. When done sorting, participants were to provide a name for each pile. Thereafter, a photo was taken of each participant’s sorting result. The same was done for the generated items of focal question 2.

#### Rating

The participants received a form containing all generated items of focal question 1 from phase 2 and were asked to rate each item in terms of *severity* on a 5-point Likert scale, where 1 indicated *not severe* and 5 *extremely severe*. The rating of these items was repeated for *difficulty to change*, where 1 indicated *very easy* and 5 *very hard* (Table 1).

**Table 1.** Description of the rating statements and rating categories for focal question 1.

Outcome	Rating statement and rating categories	Value indicator
Weight	Please rate how each item weighs for the patient: 1=Not heavy; 2=Somewhat heavy; 3=moderate heavy; 4=very heavy; 5=extremely heavy	Degree to which the item affects the patient
Change	Please rate how hard it is for the patient to change this: 1=very easy; 2=easy; 3=neutral; 4=difficult; 5=very difficult	Degree to which the item can be changed by patient

For focal question 2, the participants were asked to rate each of the generated items on 3 outcomes—importance in helping motivate patients (1 indicated *very unimportant* and 5 *very important*), acceptance by therapist (1 indicated *very unacceptable* and 5 *very acceptable*), and acceptance by patient according to therapist (1 indicated *very unacceptable* and 5 *very acceptable*; Table 2).

**Table 2.** Description of the rating statements and rating categories for focal question 2.

Outcome	Rating statement and rating categories	Value indicator
Importance	Please rate how important each item could be in motivating patients to make CBT^a^ homework: 1=very unimportant; 2=unimportant; 3=neutral; 4=important; 5=very important	Strength of perceived relationship between item and patient motivation
Acceptability therapist	Please rate how acceptable the use of each item could be for the therapist: 1=very unacceptable; 2=unacceptable; 3=neutral; 4=acceptable; 5=very acceptable	Degree to which the use of item is ethical by therapist
Acceptability patient	Please rate how acceptable the use of each item could be for the patient: 1=very unacceptable; 2=unacceptable; 3=neutral; 4=acceptable; 5=very acceptable	Degree to which the use of item is ethical by patient

a CBT: cognitive behavioral therapy.

### Phase 4: Representation

In the weeks that followed the concept mapping meeting, the data that were obtained from the previous phases were analyzed by the researchers (HML and Jeroen Ruwaard) using *The Concept System Global Max online application* [43]. The application assigns a unique number to the generated items. Several maps were generated, based on multidimensional scaling [44] with a 2D approach [45]. The software also generated the initial labels for the clusters of items. The researchers examined these cluster solutions that were considered to be logically grouped. Each potential cluster solution was discussed among the researchers. Eventually, the researchers picked 1 cluster solution that was seen as the most logical way of categorizing the generated items.

### Phase 5: Interpretation

In the interpretation phase, the generated maps were mailed to the participating therapists. They were asked whether they concurred with the proposed clusters and cluster names. When not, participants were asked to make suggestions.

### Phase 6: Utilization

After collecting the feedback from the participants in the previous phase, the researchers discussed how the data could be used to answer the research questions and made recommendations for how to use this information in future research.

### Ethical Considerations

This human participants study is compliant with the ethical rules and guidelines of the affiliated institution and has received a retrospective institutional review board exemption from the Medical Research Ethics Committee of Amsterdam University Medical Centers (review or reference 2025.0507). All participants consented to participation in the study and gave permission to use the collected data for both current and future analyses. The data collected were anonymized before analysis to protect the participants’ privacy. Participants received a gift card with a value of 15 euros (US $15.996 as per the exchange rate on March 31, 2017) as compensation for their participation.

## Results

### Phase 1: Preparation

In this study, 7 therapists (6 female and 1 male) participated in the concept mapping procedures. The concept mapping session, consisting of the generation and structuring phases, took 3 and a half hours in total.

### Phase 2: Generation

The generation phase resulted in 29 items for focal question 1 and 38 items for focal question 2.

### Phase 3: Structuring

The results of the sorting part of the structuring phase consisted of 41 piles of items for focal question 1 and 40 piles of items for focal question 2. The rating part of the structuring phase resulted in 2 forms with ratings on severity for the items generated for focal question 1 and importance for items generated for focal question 2.

### Phase 4: Representation

In concept mapping, several different maps are generated to visualize the data so that interrelationships in the information can be seen. The representation phase resulted in 15 data visualizations.

### Phase 5: Interpretation

**Figure 1.**
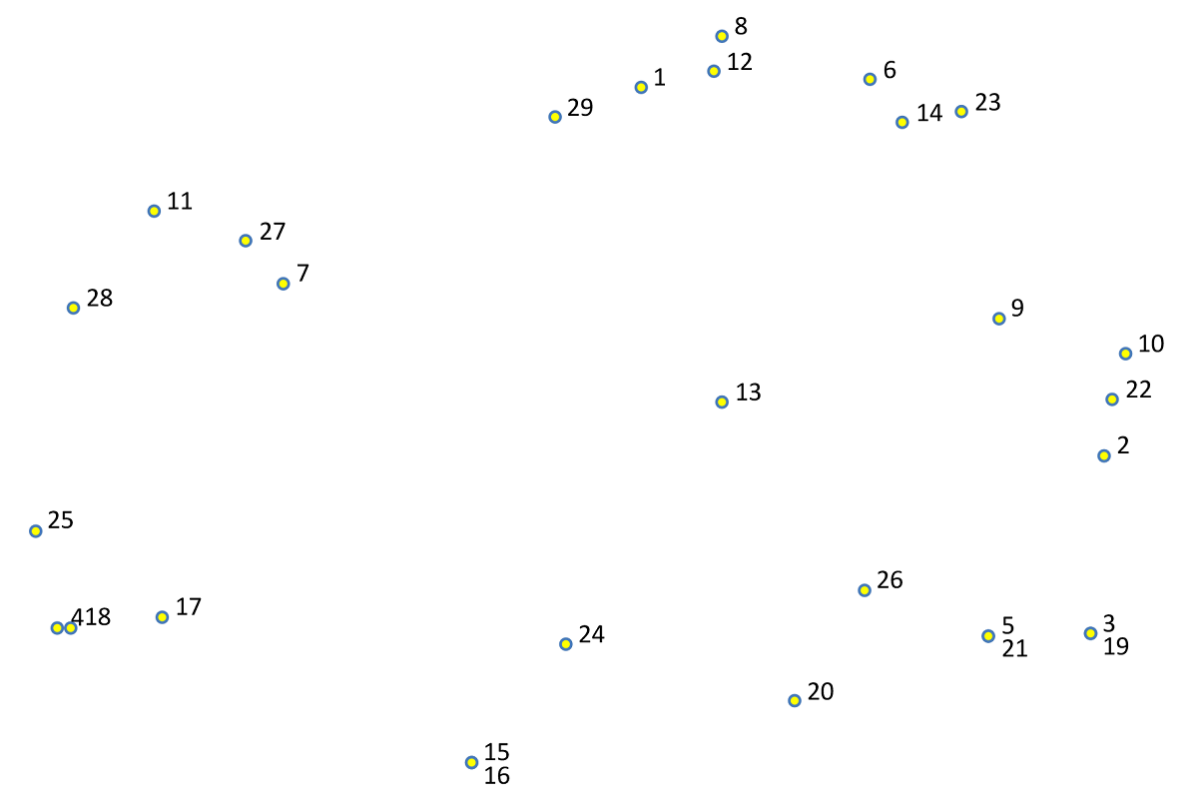
Point map of focal question 1 where distance illustrates the degree of similarity between items.

**Figure 2.**
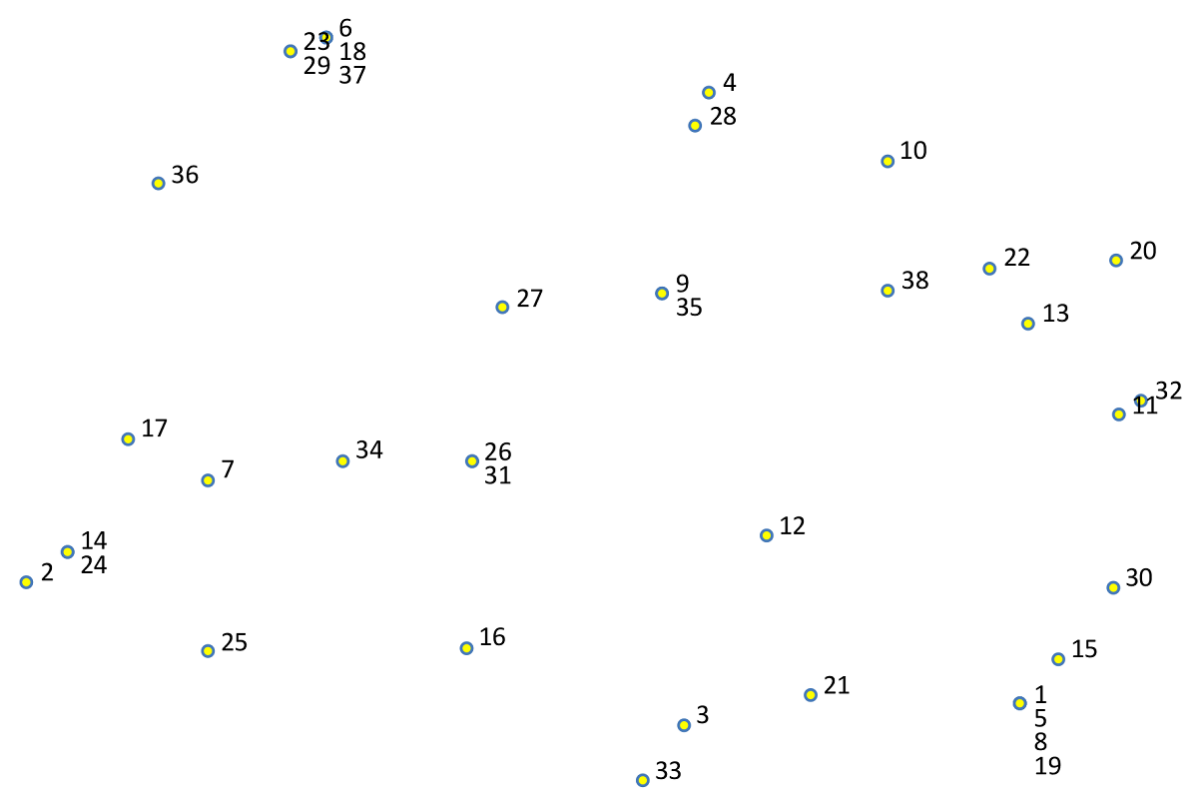
Point map of focal question 2 where distance illustrates the degree of similarity between items.

The generated maps are based on a common multidimensional scaling arranged structure named point map (Figures1  2). Each point represents a generated item. Note that the items’ exact locations on the map have no meaning. The distance between the items illustrates the degree of similarity between them. Items that are closer to each other on the map were sorted together more often by participants. For example, in Figure 1 the items 6 (boring or no fun), 14 (time consuming), and 23 (little immediate reward) were considered related by the participants as can be seen by their relative proximity on the map, while items 11 (therapist does not emphasize importance) and 20 (cognitive problems) were considered less similar by the participants and thus further away from each other. In Figure 2, items 2 (reminder), 14 (visualize pitfalls), and 24 (visualize goals) are relatively close to each other on the map and thus can be considered more similar by the participants. The distance between items 1 (rewards of different degrees), 25 (pop-ups), and 29 (invite friends) on the maps shows that the participants considered them less similar.

Tables3  4 show all of the generated items on focal questions 1 and 2, respectively, and their average ratings on homework and game element outcomes. Each item has a unique identification number. The generated items are grouped into clusters.

**Table 3.** Twenty-nine items within their 6 clusters and the average ratings for each homework outcome.

Cluster and item names (item number)	Severity rating	Difficulty rating
Homework-related		
Assignment unclear or difficult (1)	4	2.29
Boring or no fun (6)	3	2.57
Necessities not at hand (8)	3.14	1.86
Form not appealing (12)	3.29	2.14
Time consuming (14)	2.57	2.29
Little immediate reward (23)	3.29	2.29
Too much homework (29)	3.14	2
Motivation		
Too little intrinsic motivation (2)	3.86	4
Forgot to do assignment (9)	2.71	1.71
Lazy (10)	2.57	3
Not aspiring therapy goal (13)	4	3.57
Lack of discipline (22)	3.29	3.29
Symptoms related internal factors		
Fear or avoidance (3)	4	4
No self-confidence (5)	2.71	3.29
Afraid of change (19)	3.43	3.29
Cognitive problems (20)	3.43	4.29
Perfectionism (21)	3	3.57
Decrease in symptoms (26)	2.43	3.14
Symptoms unrelated internal factors		
Physically not capable (15)	3.57	4.14
Not capable because of psychological problems (16)	3.86	3.71
Trouble with Dutch language (24)	4.43	4.57
External factors		
No time due to crisis situations (4)	4	4.29
Don’t want immediate environment to know (17)	2.71	2.71
Home-situation does not provide enough space/ structure (18)	3.43	3
Therapy out of picture due to daily activities (25)	3	2.29
Therapist-related		
Different view of effect of therapy (7)	3	2.71
Therapist does not emphasize importance (11)	3.29	1.86
Doing homework only because of therapist (27)	2	2.57
Not enough reminders (28)	2.86	1.86

**Table 4.** Thirty-eight items within their 5 clusters and the average ratings for each game design element outcome.

Cluster and item names (item number)	Importance rating	Therapist acceptance rating	Client acceptance rating
Personalization			
Competition (4)	3.57	3.57	3
Aligned with interests (9)	3.57	4.57	4.43
Exclusivity (10)	2.57	3	3.57
Fantasy world (27)	3.14	3.86	3.71
Caring of figure (28)	2.86	3.57	3.43
Personalization (35)	4.14	4.14	4.14
Experience			
Unpredictable elements (11)	3.29	3.43	3.14
Beautiful lay-out (13)	4	4.86	4.71
Humour (20)	3.86	4.57	4.14
Quiz (22)	2.43	4.14	3.71
Chance element (32)	2.86	3	3.43
Stimulation of creativity (38)	3	4.43	4
Reward			
Rewards of different degrees (1)	4.43	4.86	4.71
Feedback (3)	4.14	4.29	3.86
Achievements (5)	4.14	4.57	4.43
Daily bonus that increases in value (8)	3.57	4.29	4
Time limit (12)	3	3.29	3
Unlock new features (15)	3.57	4.43	4.14
Levels (19)	4	4.57	4.43
Repetition of assignment till mastery (21)	3.43	4.14	3.43
Buy upgrades (30)	2.86	3.43	3.29
Overview of progress (33)	4	4.29	4.29
Facilitation			
Reminder (2)	4.14	4.29	3.86
Physical measurements (7)	3	3.57	3.29
Visualize pitfalls (14)	2.57	3.86	3.57
Clear end goal (16)	4.14	4.43	4.43
Daily life integration (17)	3.29	4.29	3.57
Visualize goals (24)	3.29	4.43	4.14
Pop-ups (25)	3.86	4.14	4
Short and easy (26)	3.71	4.71	4.71
Clear instructions (31)	4	5	4.71
Periodically active (34)	3.29	3	3.14
Social			
Team play (6)	2.71	3.43	3
In game social network (18)	3.29	3.86	3.29
Share with others (23)	2.86	3.14	3
Invite friends (29)	3	2.86	2.71
Therapist plays role in game (36)	2.57	3.43	3.57
Play together with experienced person (37)	4	4	3.86

**Figure 3.**
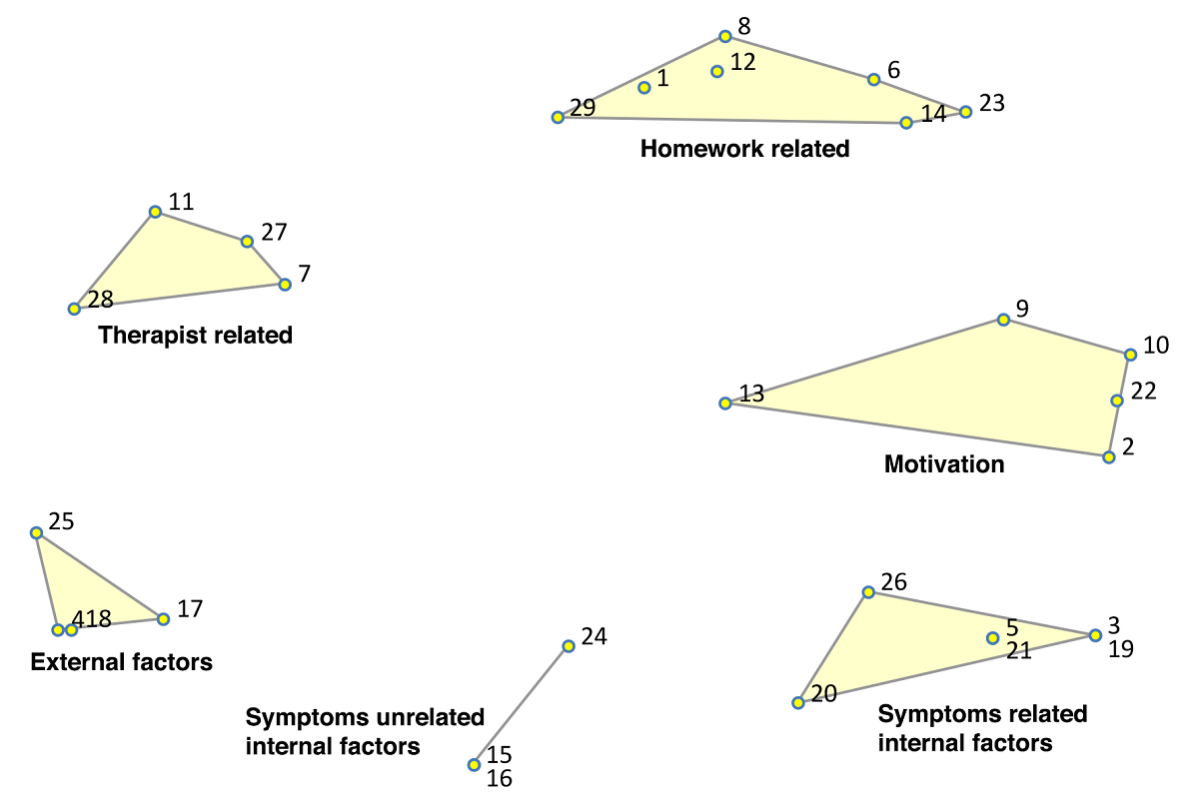
Point cluster map of focal question 1 with cluster labels chosen by researchers and participants.

In the point-cluster maps, it can be seen that a 6-cluster solution has been chosen for the reasons not doing homework, and a 5-cluster solution for the game design elements (Figures3  4, respectively). These cluster solutions were chosen by the researchers and participants based on what fitted the data best. Each cluster has a label that is the best-fit. The cluster labels were chosen by the software first. Then the researchers and participants examined the cluster labels in relation to the items in each cluster. Cluster labels that were regarded as mismatch, were replaced by new labels that the researchers and participants agreed upon.

Cluster rating maps can be used to display these ratings as well (Figures 5-9). The average ratings of all the items of the same cluster for all participants together are indicated by layers. The map of severity (Figure 5) shows that symptoms unrelated internal factors (such as, physically not capable), external factors (such as, no time due to crisis situations), and motivation (such as, not aspiring therapy goal) were rated highest and thus perceived as the most important reasons why patients do not do their homework. The map of difficulty (Figure 6) shows that symptoms unrelated and related internal factors (such as, no self-confidence) were highest in rating meaning that those factors were perceived as most difficult to change for the patient. The map of importance (Figure 7) shows that facilitation (such as, giving reminder to do homework) and rewards (such as, achievements) were perceived as most important factors that could help patients to do their homework. As an example of giving reminder, one can think of games that will give you a reminder through a notification pop-up that some tasks need to be done in order to gain points or other profits, even when the game is not opened on a smartphone. In order to use achievements to help patients do their homework, one can think of a token or digital emblem to reward the patient for doing homework. The map of acceptance therapist (Figure 8) shows that facilitation and rewards were rated highest and were thus most accepted by the therapists to implement. The maps of acceptance patient (Figure 9) shows that therapists think that facilitation and rewards will be most acceptable by patients to be used to help patients do their homework.

**Figure 4.**
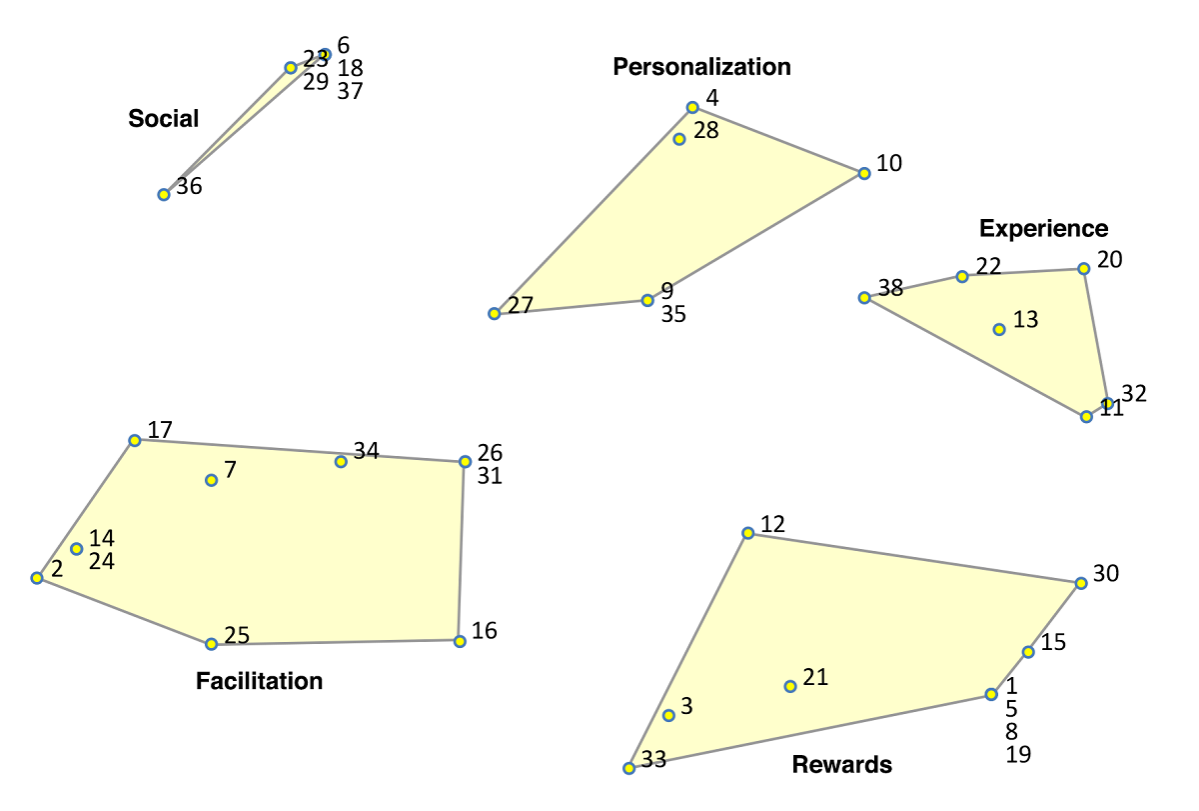
Point cluster map of focal question 2 with cluster labels chosen by researchers and participants.

**Figure 5.**
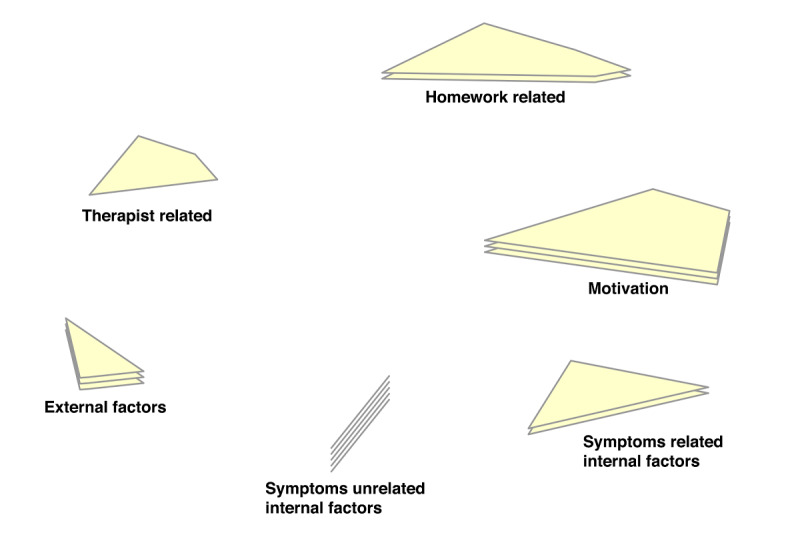
Cluster rating map of focal question 1 where the rating of severity is represented by the number of layers.

**Figure 6.**
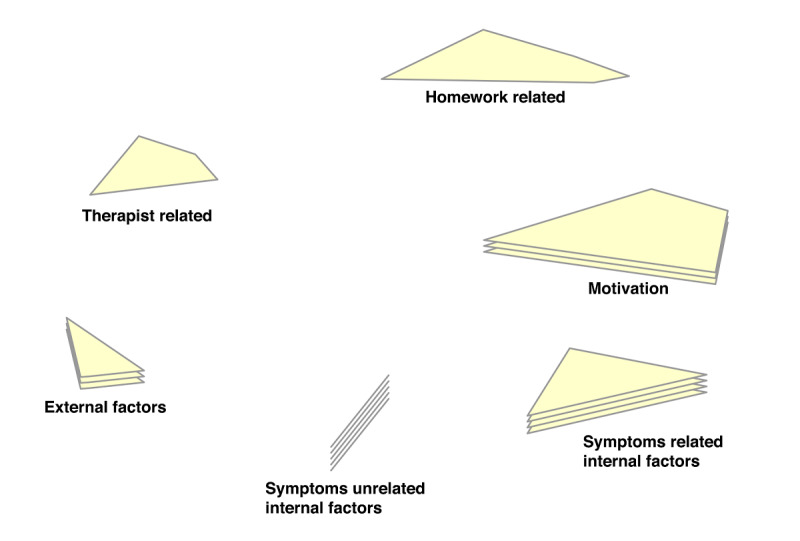
Cluster rating map of focal question 1 where the rating of difficulty is represented by the number of layers.

**Figure 7.**
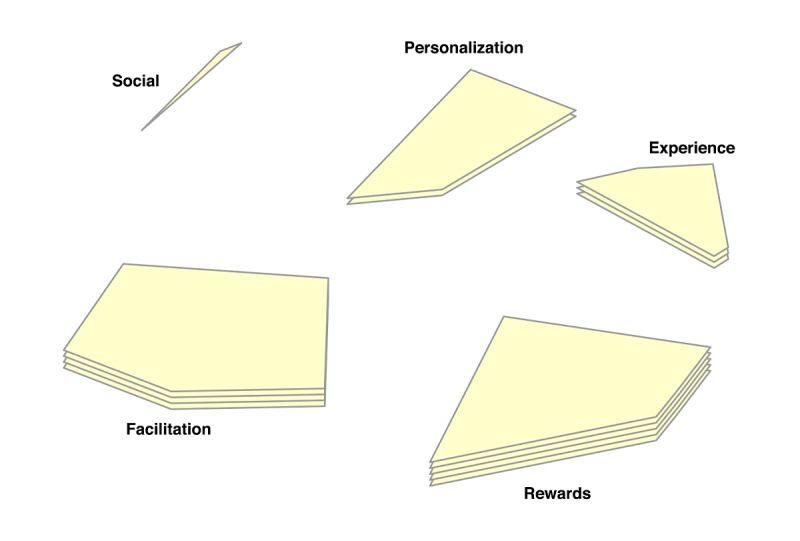
Cluster rating map of focal question 2 where the rating of importance is represented by the number of layers.

**Figure 8.**
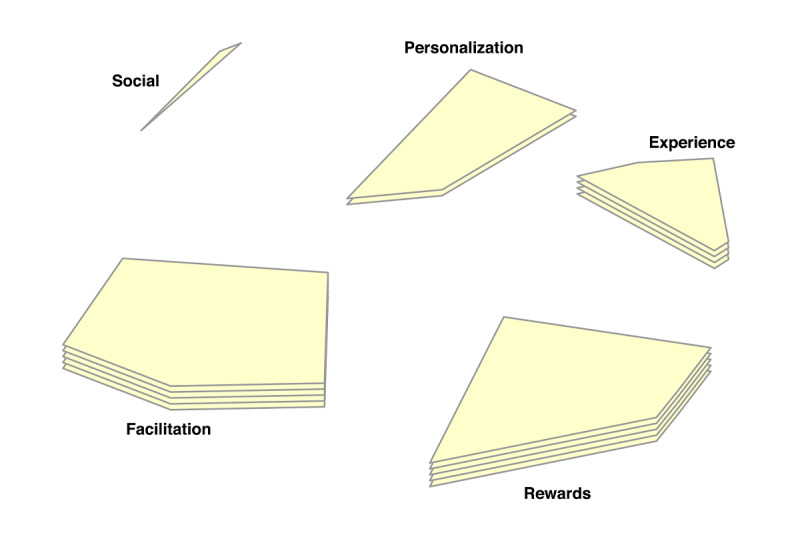
Cluster rating map of focal question 2 where the rating of acceptance by the therapist is represented by the number of layers.

**Figure 9.**
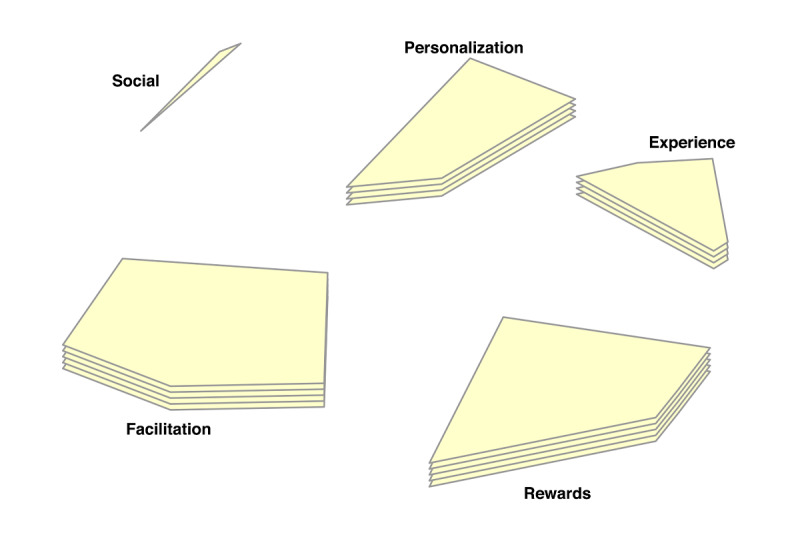
Cluster rating map of focal question 2 where the rating of acceptance by the patient is represented by the number of layers.

**Figure 10.**
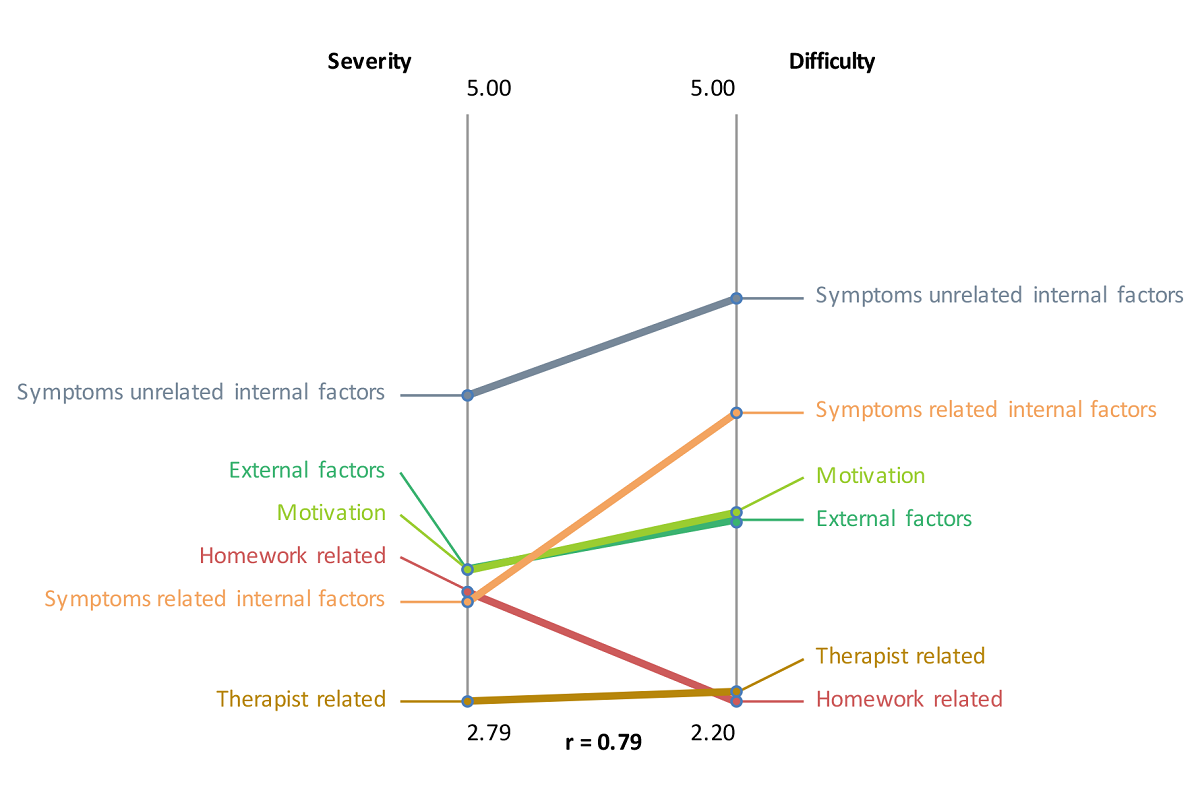
Pattern match of focal question 1 showing the relationship between severity and difficulty.

**Figure 11.**
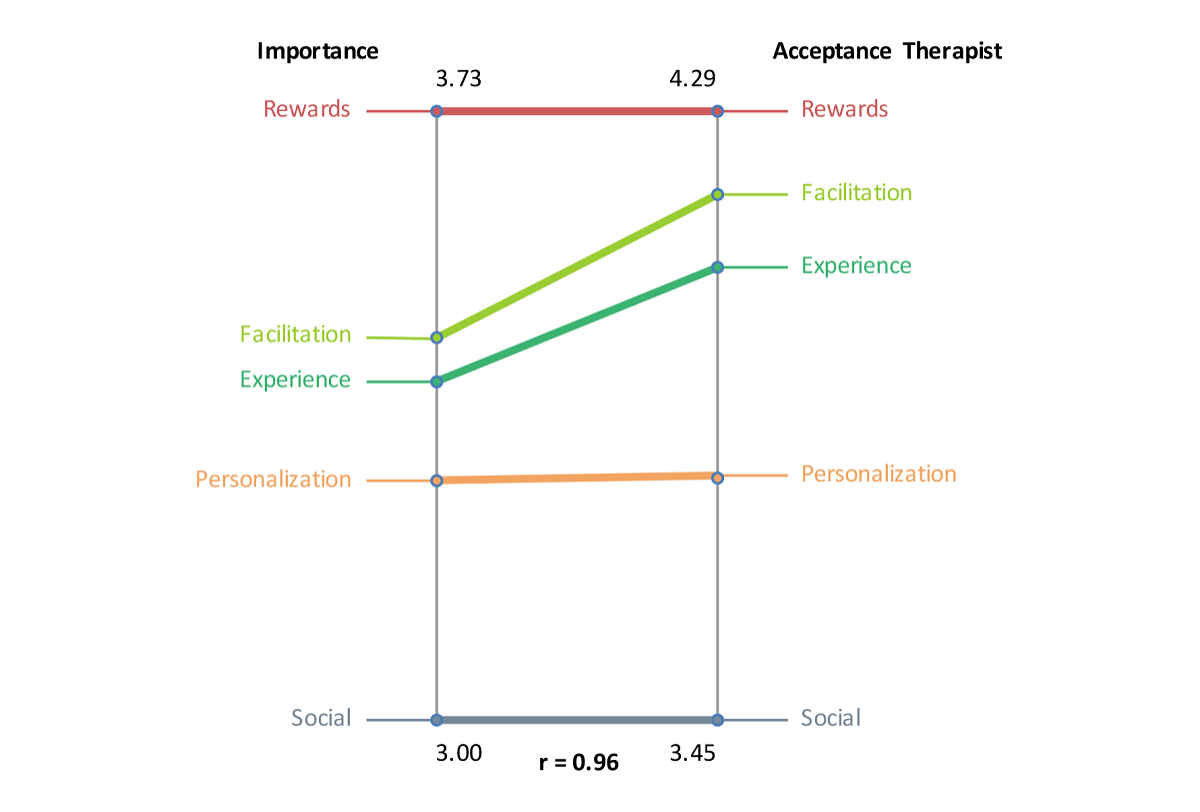
Pattern match of focal question 2 showing the relationship between importance and therapist acceptance.

**Figure 12.**
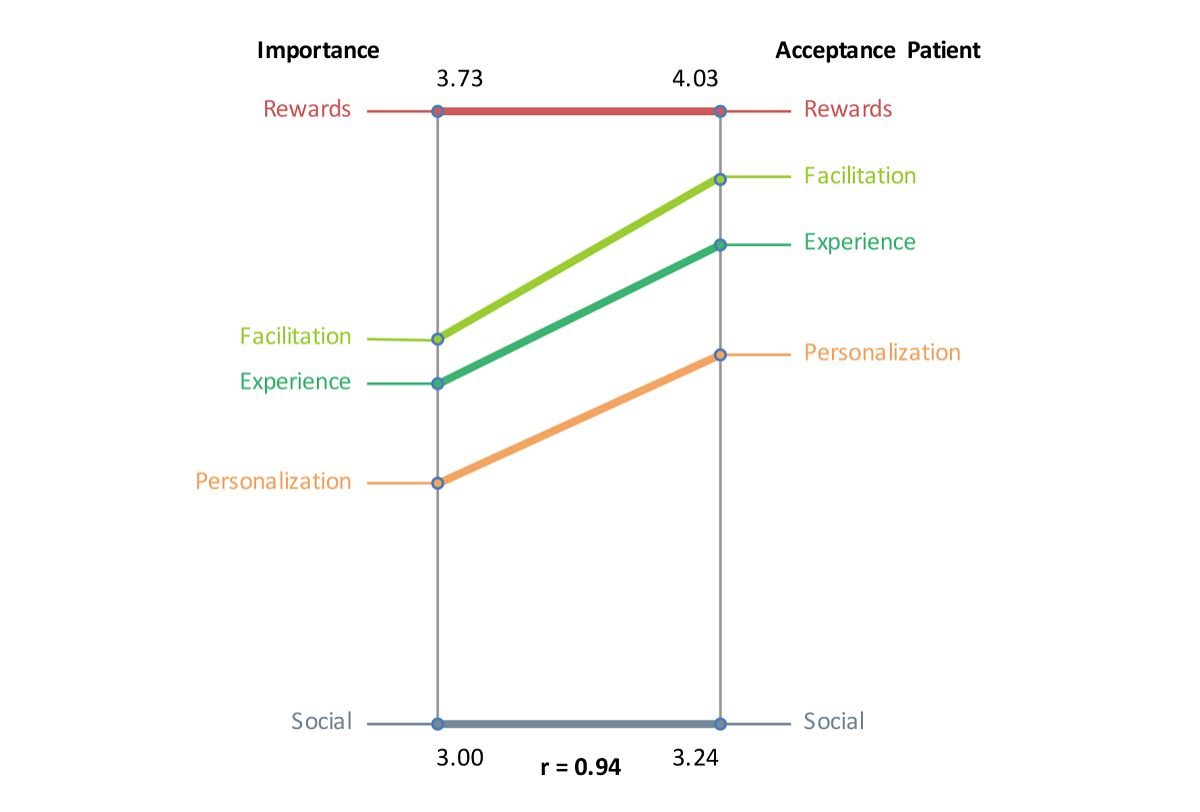
Pattern match of focal question 2 showing the relationship between importance and patient acceptance.

In pattern matching, patterns of variables are compared, as illustrated in Figures 10-12. The relationship between severity and difficulty is presented in Figure 10. Again, symptoms unrelated to internal factors (eg, physically not capable) were seen as most important for patients not to do homework. At the same time, these “symptoms unrelated to internal factors” (Figure 10) were regarded as hardest for patients to change. Homework-related reasons (eg, homework necessities not at hand) were perceived as relatively easy to change by patients. In Figure 11, the relationship between importance and acceptability of the therapist is shown. Rewards are seen as both important and acceptable by therapists. Social elements, such as inviting friends to join or an in-game social network, were perceived as not important and not acceptable by therapists due to privacy and confidentiality issues. Although facilitation and fun were acceptable to therapists, their importance was comparatively low. Figure 12 shows the relationship between importance and acceptability of the patient. The results are similar to the previous figure.

**Figure 13.**
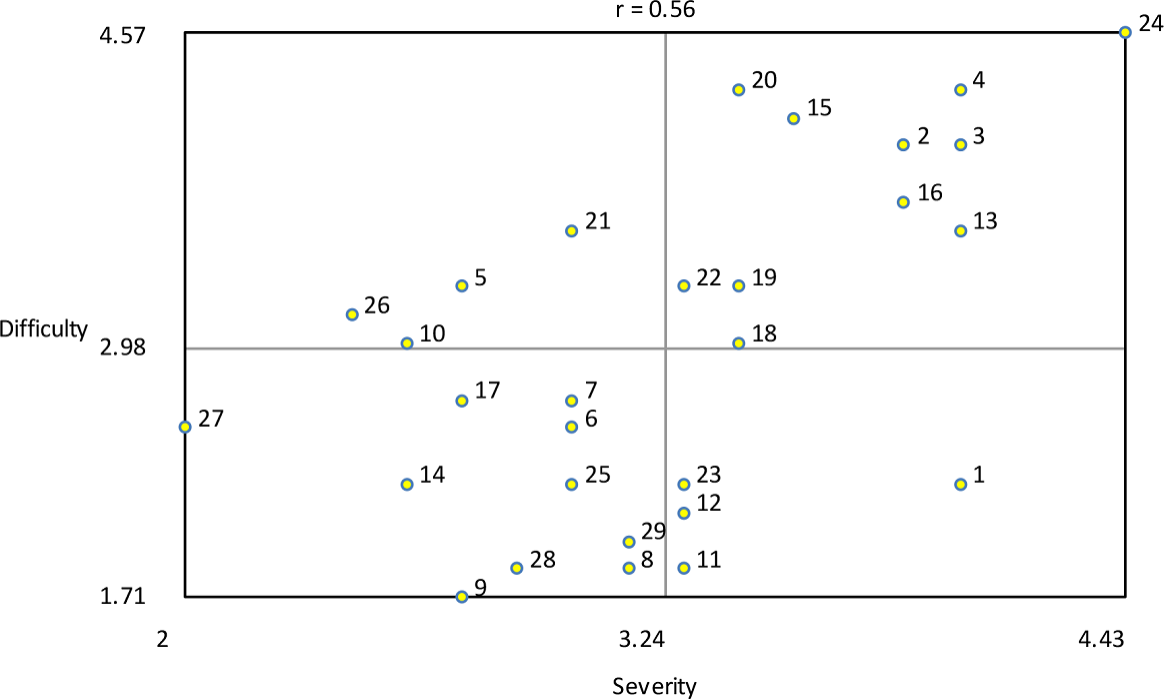
Bivariate plot of severity versus difficulty showing their relationship.

**Figure 14.**
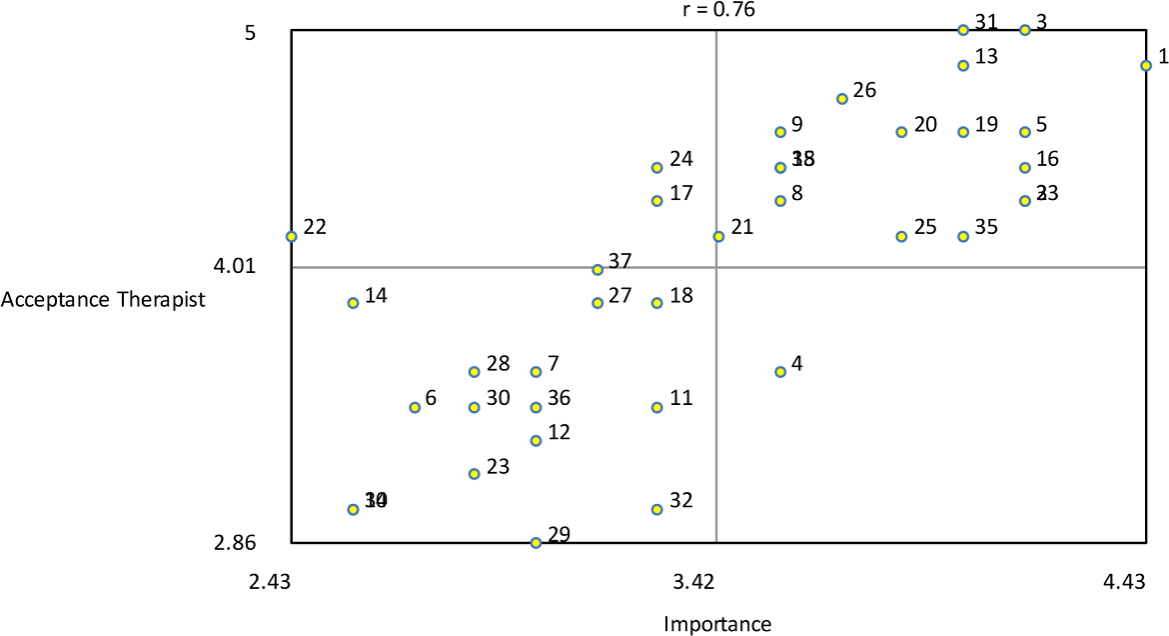
Bivariate plot of importance versus therapist acceptance showing their relationship.

**Figure 15.**
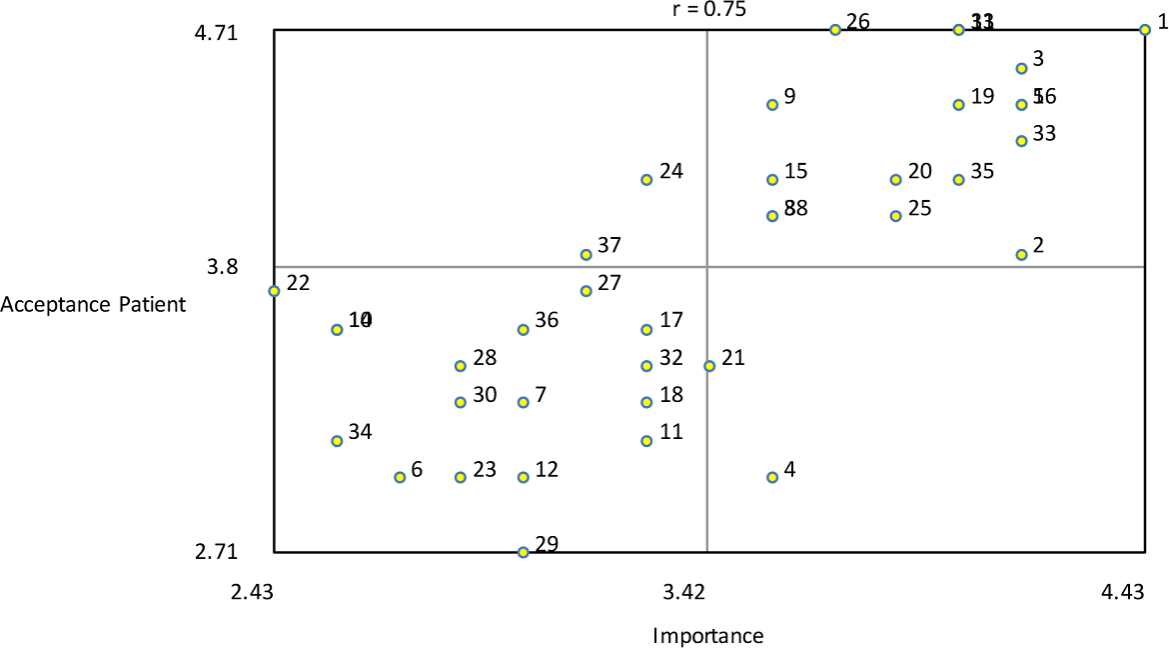
Bivariate plot of importance versus patient acceptance showing their relationship.

The variables severity and difficulty were plotted against one another to explore their relationship (Figure 13). The analysis showed that item number 24 (troubles with Dutch language) was seen as most severe and most difficult to change. The variables importance and acceptance by the therapist were plotted against one another to examine their relationship, as illustrated in Figure 14. The plot shows that game design element 1 (rewards of different degrees) was seen as the most important and one of the most accepted by therapists to implement. The variables importance and acceptance by the patient (according to the therapists) were plotted against one another to examine their relationship, as can be seen in Figure 15. The analysis showed that game design element 1 (rewards of different degrees) was seen as the most important and one of the most accepted by patients (from the therapist’s perception) to implement.

### Phase 6: Utilization

The utilization phase resulted in a summary of results, limitations, and conclusions.

## Discussion

### Summary of Results

This study aimed to gain a better understanding of the potential of using game design elements in CBT homework to boost adherence. A total of 7 behavioral therapists participated in the concept mapping session. Symptoms unrelated to internal factors (such as physical incapability) were perceived as the most important reasons for patients not to do homework. These factors were perceived as difficult to change and might not be the responsibility of the therapist. However, next to these, external factors (no time due to crisis situations, do not want immediate environment to know, home-situation does not provide enough space or structure, and therapy out of picture due to daily activities) and motivation (such as too little intrinsic motivation, forgot to do the assignment, and being lazy) were perceived as relatively important reasons that cause patients not to do assignments. These barriers are similar to the findings of Bunnell and colleagues [46]. Homework-related characteristics, although not the most important, were seen as fairly easy to change. Facilitation (such as reminders, clear end goal, daily life integration, visualize goals, pop-ups, short, and easy and clear instructions) and reward (rewards of different degrees, feedback, achievements, daily bonus that increases in value, time limit, unlock new features, levels, repetition of assignment till mastery, buy upgrades, and overview of progress) were perceived as the most important gamification clusters that could help patients do their homework. Therapists think that facilitation and rewards will be most acceptable to be used in CBT homework and also to be most accepted by patients as well.

The result of this study, the perception of therapists that the game design element facilitation, such as reminders, can help patients make CBT homework, fits nicely with earlier findings that homework adherence is higher when less time passes after the assignment of homework [10]. Giving reminders soon after the assignment of homework can thus contribute to a higher homework adherence. Also similar to earlier research, facilitation such as giving clear instructions to patients is an important component of homework adherence [47,48]. In gamified form, it could mean a tutorial or explanation of the homework with immediate rewards. In many mobile games (such as FarmVille 3 [Zynga Inc] or TopHeroes [River Game HK Limited]), the onboarding tutorial often gives immediate rewards. In the same way, using rewards, such as achievements, seems compatible with existing literature suggesting that patients’ motivation is strongly linked to homework adherence [9,12]. Earlier studies also show that using rewards in internet interventions can boost adherence, although not specifically focusing on CBT homework [35]. Although gamification seems to have potential to tackle some of the obstacles that patients could have to make CBT homework, it should be acknowledged that technology like gamification cannot solve all of these obstacles.

### Limitations

One of our limitations is the small sample size of the concept mapping, although there are no fixed rules for the sample size to be applied. Adequate sample sizes are influenced by the complexity and interactions between concepts, among others. Another limitation is the heterogeneity of the group as most of the therapists in our study were female (6 out of 7) even though it is a good representation of the field. Furthermore, no patients participated in this study; thus, the perception of what is acceptable or seen as important to implement in order to help patients do homework better cannot be confirmed.

### Conclusion

For future research, it would be interesting to study the effects of (combinations of) specific game design elements, not just the overall category or cluster. Also, there are combinations of game design elements that could cancel each other’s effects. Furthermore, the minimum number of game design elements of the same category to be used in order to be effective should also be investigated. The distinction between serving game design elements online or traditionally (nondigitally) is another intriguing aspect. Furthermore, research on patients’ preferences on to be implemented game design elements is needed. All these could contribute to the future of using gamification to help patients do their CBT homework better and thus improve therapy outcomes.

## Abbreviations

CBT: cognitive behavioral therapy
